# Convergent evolution of disordered lipidic structural colour in the fruits of *Lantana strigocamara* (syn. *L. camara* hybrid cultivar)

**DOI:** 10.1111/nph.18262

**Published:** 2022-06-10

**Authors:** Miranda A. Sinnott‐Armstrong, Yu Ogawa, Gea Theodora van de Kerkhof, Silvia Vignolini, Stacey D. Smith

**Affiliations:** ^1^ Department of Chemistry University of Cambridge Lensfield Road Cambridge CB2 1EW UK; ^2^ Department of Ecology & Evolutionary Biology University of Colorado‐Boulder Boulder CO 80309 USA; ^3^ Univ. Grenoble Alpes, CNRS, CERMAV Grenoble 38000 France

**Keywords:** convergent evolution, fruit colour, honest signalling, lantana, plant lipids, seed dispersal, structural colour

## Abstract

- The majority of plant colours are produced by anthocyanin and carotenoid pigments, but colouration obtained by nanostructured materials (i.e. structural colours) is increasingly reported in plants. Here, we identify a multilayer photonic structure in the fruits of *Lantana strigocamara* and compare it with a similar structure in *Viburnum tinus* fruits.
- We used a combination of transmission electron microscopy (EM), serial EM tomography, scanning force microscopy and optical simulations to characterise the photonic structure in *L. strigocamara*. We also examine the development of the structure during maturation.
- We found that the structural colour derives from a disordered, multilayered reflector consisting of lipid droplets of *c.*105 nm that form a plate‐like structure in 3D. This structure begins to form early in development and reflects blue wavelengths of light with increasing intensity over time as the structure develops. The materials used are likely to be lipid polymers.
- *Lantana strigocamara* is the second origin of a lipid‐based photonic structure, convergently evolved with the structure in *Viburnum tinus*. Chemical differences between the lipids in *L. strigocamara* and those of *V. tinus* suggest a distinct evolutionary trajectory with implications for the signalling function of structural colours in fruits.

## Introduction

Colour diversity in plants is derived from two primary mechanisms: pigments (such as anthocyanins, carotenoids, and betalains) and structural colours (such as diffraction gratings, multilayer reflectors, or photonic crystals) (Glover & Whitney, 2010; Vignolini *et al*., 2013). Structural colours result from the interaction of light with nanostructures similar in size to wavelengths of visible light (Kinoshita & Yoshioka, 2005). In animals, both pigments and structural colours are common and widely reported. Most blues and many greens in birds, butterflies and beetles – as well as blue skin and scales in vertebrates – are structural colours (Bagnara *et al*., 2007). However, structural colours have been less studied in plants. Only a few types of architectures have been observed from a handful of species with structurally coloured leaves, flowers or fruits (e.g. Vignolini *et al*., 2012b; Strout *et al*., 2013; Jacobs *et al*., 2016; Moyroud *et al*., 2017). Recent work has expanded our understanding of structural colours in plants, including the characterisation of novel structures in fruits (Middleton *et al*., 2020), the description of convergent evolution of floral photonic structures across diverse plant clades (Moyroud *et al*., 2017), and the discovery of a variety of structures that produce optical effects that enhance pigmentary colouration (Vignolini *et al*., 2012a,c; Wilts *et al*., 2018).

Among diaspores (fruits and seeds) dispersed by animals, only five species with structural colours have been described to date. *Pollia condensata* and *Margaritaria nobilis* both produce helicoidal cellulose nanofibers, which are arranged like a spiral staircase and reflect polarised blue light (Vignolini *et al*., 2012b, 2016). Two additional species, *Elaeocarpus angustifolius* and *Delarbrea michieana*, have multilayered reflectors, although the materials used to build these reflectors remain undescribed (Lee, 1991; Lee *et al*., 2000). Finally, *Viburnum tinus* was recently described as having a disordered multilayer reflector consisting of small lipid globules arranged in periodic layers (Middleton *et al*., 2020).

**Fig. 1:**
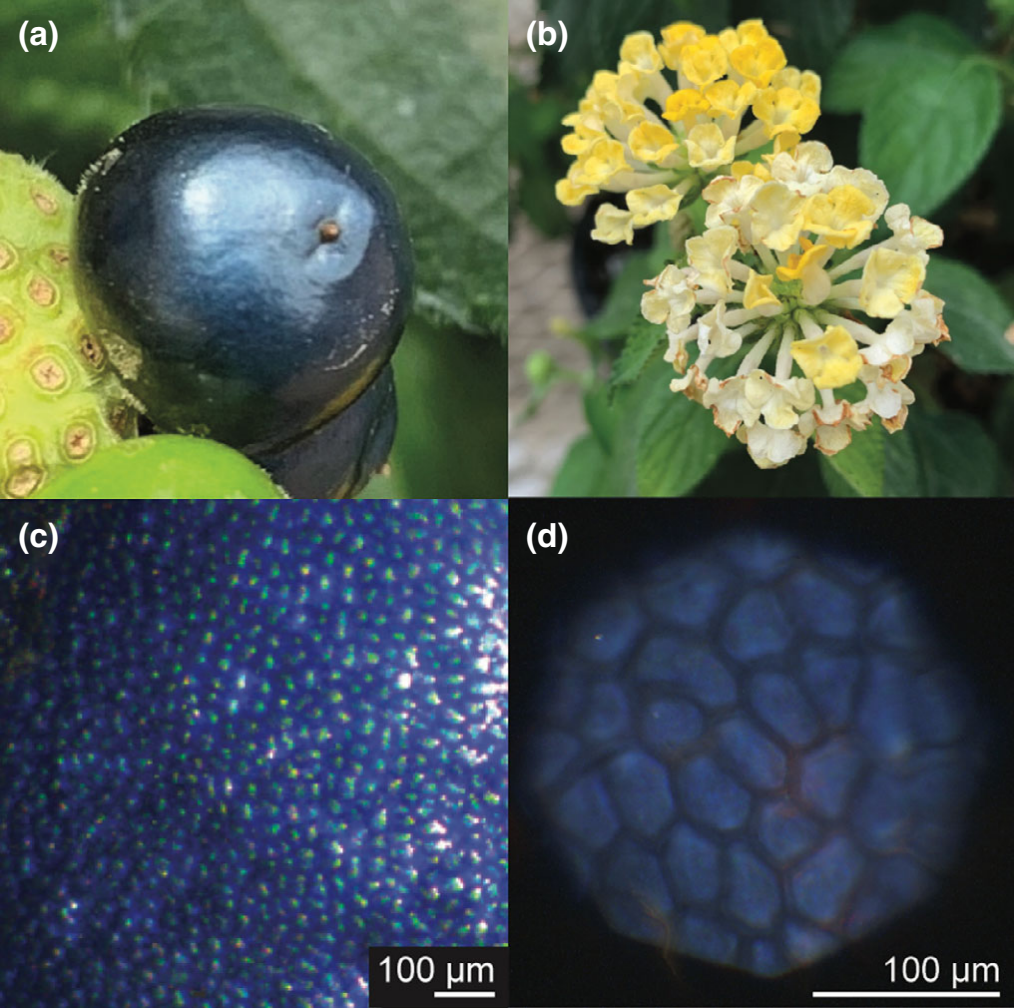
*Lantana strigocamara* has metallic blue fruits. (a) Photograph of mature *L. strigocamara* fruit, *c*. 5 mm in size. (b) Flowers of *L. strigocamara*. (c) Microscopic image of surface of *L. strigocamara* fruit. (d) Image taken under parallel polarising filters showing blue colouration visible at a microscopic scale. Images taken under crossed polarising filters reflect almost no light (see Supporting Information Fig. S2) and therefore appear entirely black and are not depicted here.

Here, we report a new species with structurally coloured fruits, *Lantana strigocamara* (syn. *L. camara* hybrid cultivar), which produces small (*c*. 5 mm in diameter; Fig. 1a), metallic blue fruits with a photonic structure with highly convergent morphology to the multilayer reflector in *V. tinus. Lantana strigocamara* is a commonly planted horticultural herb or shrub (Fig. 1b) that is highly invasive around the world (Sharma *et al*., 2005; Bhagwat *et al*., 2012), causing economic losses for local communities (Sundaram *et al*., 2012; Shackleton *et al*., 2017), reducing frugivore diversity in some habitats (Aravind *et al*., 2010), and threatening native *Lantana* species through introgression (Maschinski *et al*., 2010). We characterised the nano‐architecture responsible for the structural colour in *L. strigocamara*, compared it with *V. tinus*, and discussed the implications for the evolution and ecology of structurally coloured fruits.

## Materials and Methods

### Plant collection

We grew *Lantana strigocamara* plants, acquired from horticultural sources, in the University of Colorado‐Boulder glasshouses and deposited a voucher collection at the University of Colorado Herbarium (K. Dodson 003 (COLO)). We sampled fruits throughout a developmental sequence ranging from fruits barely post‐fertilisation through to mature stages when the blue colour was present (Fig. 1a). To collect this sequence, we selected fruits across different size classes as a proxy for developmental age.

### Optical characterisation

To quantify fruit colour at the macroscale, we measured reflectance using a Jaz spectrometer (OceanOptics, Dunedin, FL, USA) equipped with a deuterium–halogen lamp and a ultraviolet (UV) detector, and a fibre core size of 450 μm. We used an anodised aluminium probe holder to eliminate background light and to hold the probe at 45° consistently across samples. Finally, we calibrated the reflectance measurements against a Spectralon white standard (Labsphere, North Sutton, NH, USA). We used a stereoscope (Stemi 305; Carl Zeiss AG, Jena, Germany) with side illumination to obtain the micrograph in Fig. 1c. For the image in Fig. 1d, we used a ZEISS Axio Scope A1 with a charged coupled device (CCD) camera (IDS‐UI‐3580LE; IDS Imaging Development Systems, Obersulm, Germany) in reflection mode, with a Zeiss EC Epiplan Apochromat objective (422652‐9960). For microspectrometry, light was coupled from the Zeiss AxioScope via an optical fibre (100 μm internal diameter) to a spectrometer (AvaSpec‐HS2048; Avantes BV, Apeldoorn, the Netherlands). The spectra were referenced against a silver mirror (PF10‐03‐P01). For polarisation measurements, two WP25M‐UB linear polarisers were used, one for incident light and the other for reflection.

### Transmission electron microscopy

We fixed 2 mm^2^ sections of fruit epicarp tissue in cacodylate‐buffered 2% glutaraldehyde fixative. For embedding, we first stained with 1% osmium tetroxide for 2 h, dehydrated through an ethanol series, and finally infiltrated overnight in 50% ethanol : 50% Epon resin followed by resin changes every 24 h for 3 d. To polymerise, we added DMP accelerator and polymerised the samples at 60°C for 24 h (Supporting Information Table S1). We used a 35° diamond knife on a Leica UCT ultramicrotome to cut 80‐nm ultrathin sections, which we mounted on copper mesh grids and imaged on an FEI Tecnai T12 transmission electron microscope (TEM). Measurements of cell wall components (cuticle, layered photonic structure, and primary cell wall) were performed in fiji (Schindelin *et al*., 2012) for 4–13 cells per developmental stage.

### Optical simulations

We simulated 2D layered structures and then modelled their optical response using finite difference time domain calculations (FDTD) in a commercial software (Lumerical FDTD Solutions; v.211, http://www.lumerical.com), using code adapted from Middleton *et al*. (2020). We tested whether the optical parameters (layer thickness and periodicity) of the photonic structures observed in *L. strigocamara* could reflect short wavelengths similar to the measured reflectances. We altered the *Viburnum tinus* parameters (layer thickness: 88 nm; periodicity: 190 nm) to match those measured from *L. strigocamara* cross‐sectional images (layer thickness: 105.3 nm; periodicity: 110.7 nm) and retained the same degree of angular disorder (sigmaPhi = 6). Five replicate multilayered structures were generated to consider the inhomogeneity of the structure and the average simulated reflectance was reported.

### Serial tomography

We embedded the samples as described above, cut three serial thick sections (300 nm), placed them on Formvar‐coated slot grids, and then marked both sides with 15‐nm gold fiduciary markers. We performed a single‐axis tilt series (from −60° to 60°, imaging every 1°) on an FEI Tecnai F30 TEM at 300 kV. We joined the serial section images together using iMod (Kremer *et al*., 1996), which we also used to create 3D isosurface reconstructions of the photonic structure.

### Chemical characterisation

To chemically characterise the photonic structure, we applied a similar method to that described in Sinnott‐Armstrong *et al*. (2020b): we cryo‐cut ultrathin sections of fresh fruit material, imaged them in a JEM 2100Plus TEM (Jeol, Tokyo, Japan), exposed them to various solvents, and then re‐imaged them to visualise the effect of the solvents. We initially tried chloroform, which extracted the globules in *V. tinus* but caused only minor changes in the globular structure in *L. strigocamara* (Fig. S1). We then tested ethanol, acetone and water, with little visible change in the electron density of the globules. Finally, we hydrolysed samples following the approach in Veličković *et al*. (2014) by exposing the ultrathin sections of fruit tissue to 0.15 M KOH in dry ethanol.

### Mechanical properties

We performed scanning force microscopy (SFM) using a Dimension ICON instrument (Bruker) in a pulsed force (PeakForce) mode to compare the mechanical properties of the cell wall, photonic structure and cuticle.

### Nutrition

We collected pulp from *L. strigocamara* fruits that we dried for later chemical analysis. We pulverised the pulp and analysed lipid content using an Ankom XT‐15 Extractor (Ankom Technology, Macedon, NY, USA). In brief, we weighed three replicates of 1.5 g each into acid‐free filter bags. Then, we exposed the tissue to petroleum ether at 90°C and weighed mass pre‐ and post‐extraction with petroleum ether. Crude fat content was calculated as (pre‐extraction – post‐extraction)/(pre‐extraction) to produce a percentage of dry weight.

## Results

### Optical characterisation

Reflectance of *L. strigocamara* fruits increases towards shorter wavelengths with a peak at 377 nm. The microspectrometric measurements showed that reflectance is high under parallel polarising filters but nearly zero under crossed polarising filters (Fig. S2), indicating that the polarisation of the light is conserved upon reflection from the fruit skin. This observation clearly demonstrates a structural component to the colouration.

### Nanostructural architecture

**Fig. 2:**
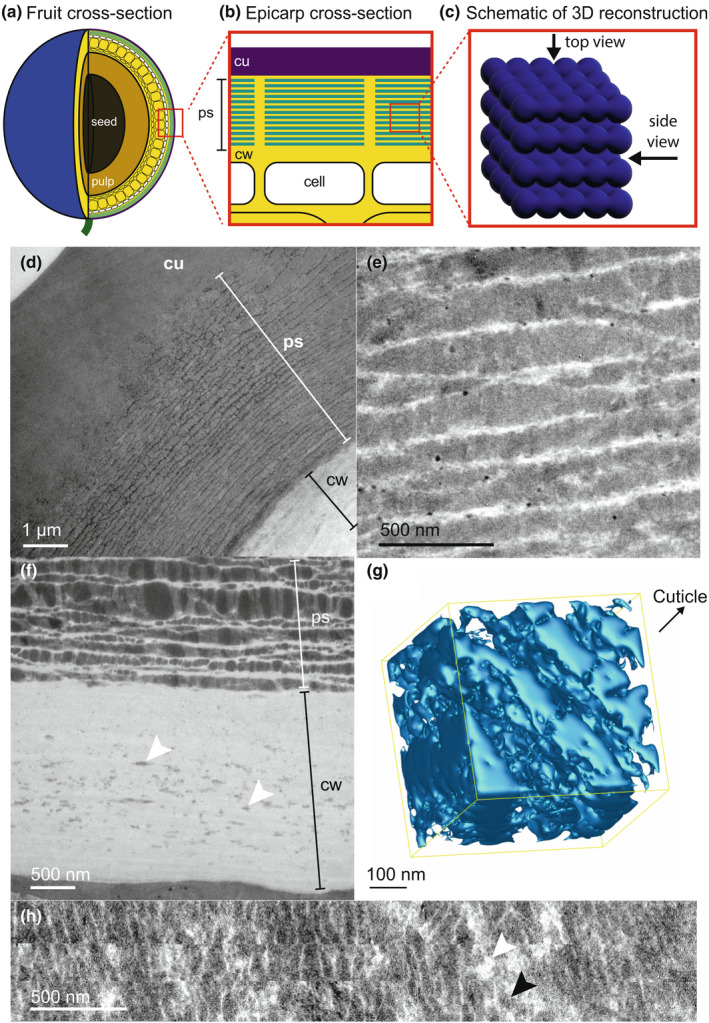
Nanostructural morphology of the cell wall of *Lantana strigocamara* fruits. (a) Illustration of a cross‐section of a fruit indicating the epicarp cells (in white) with additional cell layers (in yellow), pulp (light brown) and the seed in the centre of the fruit (dark brown). (b) The photonic structure occurs in the cell wall of the epicarp cells and appears as a stack of layers. (c) A schematic representation of the 3D structure of the stack of layers, shown from a cross‐sectional (side) view and from a top view. Transmission electron microscope (TEM) images show (d) the full structure and (e) a higher magnification image of the layered photonic structure. (f) Small globules embedded in the cell wall (white arrows) appear to be released from the cytoplasm, traverse the cell wall and then compact and merge with other lipidic globules to form the layered structure. Isosurface reconstructions of serial electron microscopy tomograms reveal the 3D architecture of the photonic structure. (g) A cross‐section of the serial tomogram shows the alternation of plate‐like layers. Please refer to Supporting Information Video S1 for video rotation of this reconstruction. (h) A top view of the photonic structure, constructed from the serial tomogram, shows that the globules form relatively complete plate‐like layers in the cell wall, although they are not perfectly continuous. Dark patches (black arrowheads) indicate lipid globules, light patches (white arrowheads) indicate cell wall matrix. *cu*, cuticle; *cw*, cell wall; *ps*, the photonic structure.

Using TEM, we observe a layered structure in the epicarp cell walls of *Lantana strigocamara* fruits (Fig. 2a–c). Each layer was *c*. 105 nm thick with a mean periodicity of 110 nm (Fig. 2d,e). Small globules occur in the primary cell wall and appear to migrate through the cellulose‐rich region to compact against the bottom layer of the structure (Fig. 2f). Tomographic reconstructions revealed that this structure forms plate‐like layers in 3D (Fig. 2g; Video S1). A rotated view of the structure (Fig. 2h) shows a cross‐section of an example layer, illustrating that each layer is plate‐like but not perfectly continuous.

### Optical simulations

Our simulations produced a peak reflectance at 340 nm (Fig. S3), indicating that a layered structure such as that in *L. strigocamara* could reflect short wavelengths that are congruent with the observed reflectance of short wavelengths from *L. strigocamara* fruits. The location of the peak from simulations depends on various factors, including layer thickness and periodicity, which contribute to differences between the simulated and observed reflectances. Nonetheless, our model captures the primary effect of short‐wave reflectance from the layered photonic structure.

### Developmental trajectory

**Fig. 3:**
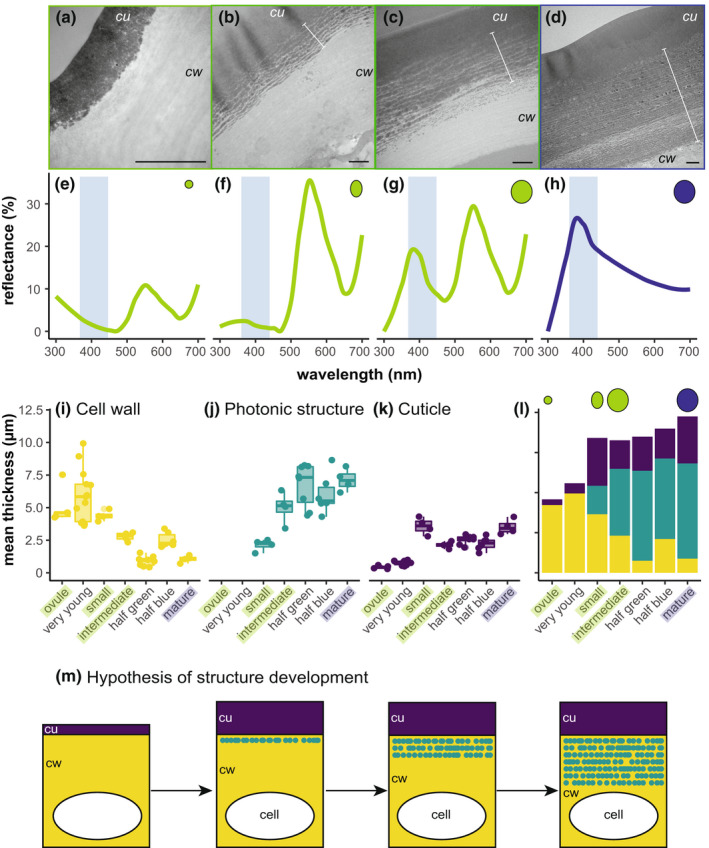
Developmental series of *Lantana strigocamara* fruits demonstrates that the lipidic structure begins to accumulate early in fruit development, although not immediately after fertilisation. (a–d) Transmission electron micrographs of the cell walls of *L. strigocamara* fruits during a developmental series, beginning with (a) a fertilised ovule still inside the senesced flower, to (b) a small fruit post‐floral dehiscence, to (c) a green but full‐size fruit, to (d) a mature blue fruit. *cu*, cuticle; *cw*, cell wall. (e–h) Reflectance measurements of the fruit depicted in the transmission electron microscope (TEM) images in (a–d), (e) fertilised ovule, (f) small fruit, (g) green full‐size fruit, and (h) mature blue fruit. Reflectance measurements illustrate this process at the macroscale, in which reflectance in the blue region (< 450 nm) increases with increasing developmental maturity. Note that the peak at *c*. 550 nm in the three green stages is due to chlorophyll. White bars delineate photonic structures in (b, c and d) (there is no photonic structure in (a)). Bar, 1 μm in all images. Boxplots of measurements of cell wall regions ((i) primary cell wall, (j) photonic structure, and (k) cuticle) illustrate the change in region thickness across seven fruit developmental stages. Points represent measurements from individual cells, horizontal bars indicate the median value, and points outside of the whiskers are outliers. Highlighted labels indicate stages corresponding to those illustrated by TEM images in (a–d). (l) Histogram showing the change in thickness of different regions of the cell wall across development, including (from top to bottom) cuticle, photonic structure and primary cell wall regions. Measurements in (i–l) are based on at least four individual cells per developmental stage. (m) Schematic illustrating the hypothesis of development of the cell wall. Purple, cuticle; teal, photonic structure; yellow, primary cell wall.

Across development, globules accumulate in a layered structure over time (Fig. 3a–d) that corresponds with increasing reflectance of blue wavelengths of light (*c*. 400–450 nm; Fig. 3e–h). Development begins with a thick primary cell wall, no layers, and a thin cuticle (Fig. 3a). As the fruit matures, the primary cell wall becomes thinner (Fig. 3i) and the globular region and the cuticle both grow in thickness (Fig. 3j,k). Overall, most of the cell wall is laid down early, and then transitions to a multilayered structure as lipid layers accumulate, converting the primary cell wall to the globular region (Fig. 3l,m).

### Chemical characterisation

**Fig. 4:**
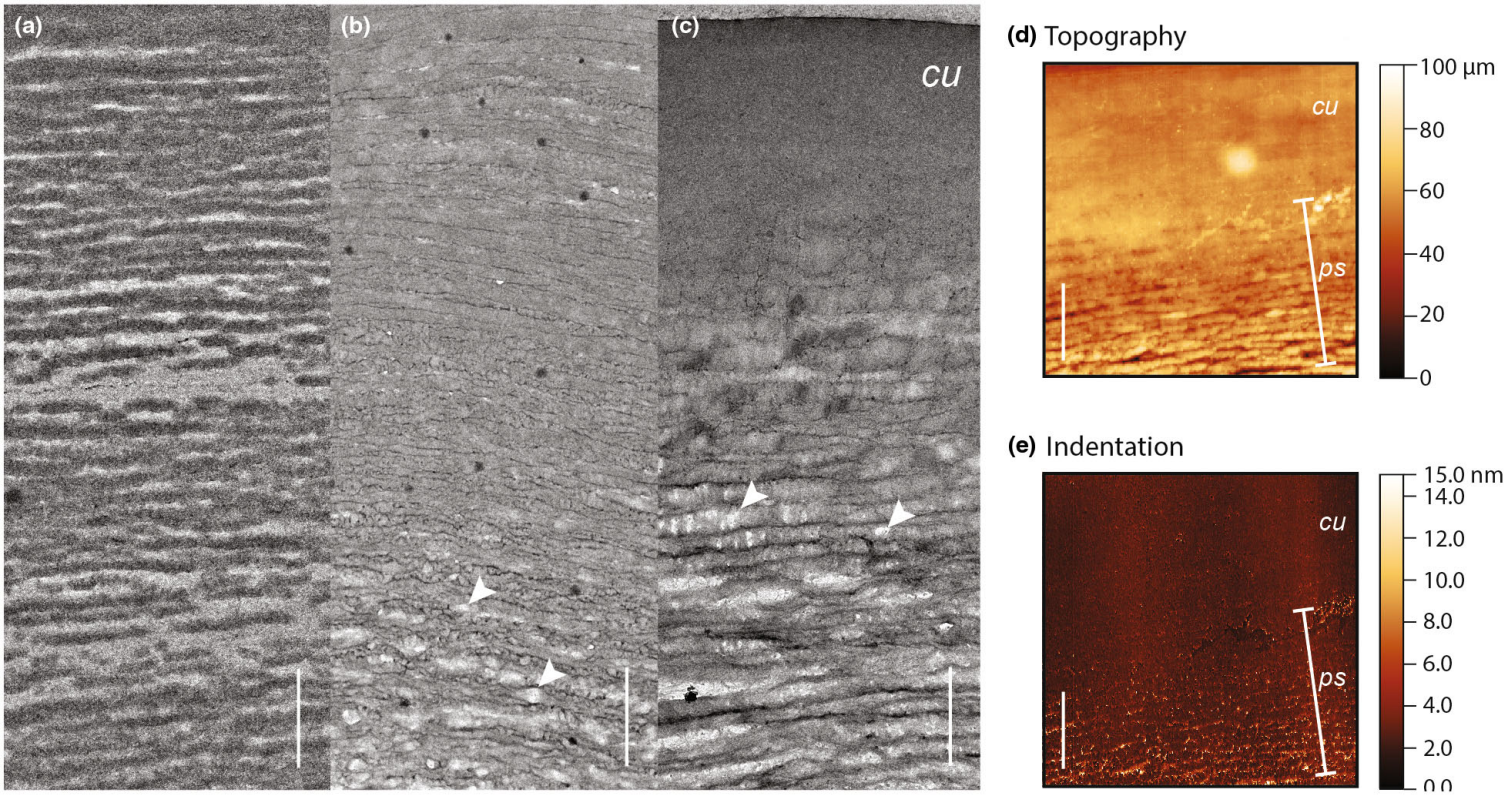
Chemical composition and mechanical properties of the globules in *Lantana strigocamara* are consistent with the hypothesis that the globules are similar to the cuticle. Globules are soluble in a mixture of potassium hydroxide (KOH) and ethanol (EtOH), indicating that extracted compounds are lipids, and that the globule materials are likely to be a mixture of a variety of compounds including waxy polymers. (a) Pre‐extraction globules are present. Dark areas are the globules and cuticle, lighter areas the matrix between layers of globules. (b) Following a 10‐min exposure to KOH and EtOH, some globules were extracted, especially those closest to the cytoplasm and farthest from the cuticle. (c) Increasing exposure to KOH and EtOH for 15 min in total leads to increasing extraction especially adjacent to the cuticle. In (b, c) white arrowheads indicate extracted globules. Mechanically, the globules also resemble the cuticle: scanning force microscopy (SFM) confirms that the globules are similar to the cuticle in both (d) topography (the height of the sample) and (e) indentation behaviour (how far into the sample the probe tip penetrates). Bar, 1 μm in all images, *cu*, cuticle; *ps*, photonic structure.

Exposure to a dilute mixture of KOH + EtOH resulted in increasing loss of globules with increasing time of exposure (Fig. 4a–c). Consequently, we inferred that the hydrolysed products of the globules were highly soluble in ethanol, which indicated that they were lipidic in nature, and likely to be waxy polymers whose monomers (free fatty acids) are soluble in ethanol. The globules forming the multilayered structure did not extract in chloroform (Fig. S3).

### Mechanical properties

SFM images indicated that the globules share mechanical properties with the cuticle, in particular deformation behaviours (Fig. 4d,e). These results suggest a similar chemical composition of the cuticle and globules relative to the cellulosic matrix as the deformation behaviour reflects the hardness of the material and therefore its chemical composition.

### Nutrition

*Lantana strigocamara* fruits have a very low lipid nutritional value, < 1%.

## Discussion

We observed a disordered, multilayered photonic structure in the epicarp cell wall of *Lantana strigocamara* fruits. This structure was highly convergent, morphologically and phenotypically, with the structure previously described in *Viburnum tinus* (Middleton *et al*., 2020). Both species have metallic blue fruits with similar disordered, multilayered photonic structures in their epicarp cell walls. Serial tomography revealed that both species produced a 3D stack of plate‐like layers, embedded in a cell wall matrix. *V. tinus* and *L. strigocamara* belong to different major clades of angiosperms (Dipsacales and Lamiales, respectively) who last shared a common ancestor more than 100 million years ago (Ma) (Stevens, 2001; timetree.org). These photonic structures clearly evolved convergently, making *L. strigocamara* only the second origin of a disordered, multilayer structural colour made out of lipidic molecules in plants and, indeed, in any organism known to date.

### Evolutionary origins of the photonic structure in *L. strigocamara*

This marked convergence occurred solely at the morphological and phenotypic levels. Chemically, these structures are different in ways that may impact their ecological function. In *V. tinus*, the lipid globules were readily extractable by chloroform and are likely to be low‐molecular‐weight fatty acids (Middleton *et al*., 2020). By contrast, the globules *L. strigocamara* were not soluble in chloroform, but hydrolysis resulted in lipids that were soluble in ethanol. These findings suggested that the globules in *L. strigocamara* are polymers of polar lipids. The differing chemistry of the globules in *L. strigocamara* relative to those in *Viburnum tinus* indicates that the structures in the two species are likely to have different evolutionary/developmental origins. Extracellular lipids are common and diverse in plants, and range from the cuticle itself, to triglycerides embedded in the cell wall, to exosomes of various kinds (Chapman *et al*., 2012; Horn & Chapman, 2012; Rutter & Innes, 2018).

The results of our chemical extractions and SFM measurements are consistent with a cuticle‐related origin of the structure in *L. strigocamara*, although we emphasise that this is still speculative as the exact chemistry of the extracted lipids remains unknown. Typically, cuticular waxes (but not cutin) are soluble in chloroform (Jeffree, 2006), and the lack of dissolution of the globules forming the photonic structure suggests that they are neither waxes nor low‐molecular‐weight fatty acids. Chemically, the cuticle consists of a variety of compounds, including diverse waxy polymers (Riederer & Muller, 2006; Yeats & Rose, 2013). The compounds in *L. strigocamara* globules are lipid polymers, and our extractions of lipids in *L. strigocamara* are more successful for those globules closer to the cell (Fig. 4) suggesting that the globules may become increasingly polymerised as they migrate through the cell wall. Additionally, the mechanical properties of the globules match those of the cuticle, although similar mechanical properties alone cannot confirm whether the globules are cutin or cuticle related. Morphologically, the globular structure in *L. strigocamara* appears to merge with the cuticle (e.g. Fig. 2). Globules of cuticle material of various sizes are well known in the literature (e.g. Buda *et al*., 2009; Martin & Rose, 2014; Domínguez *et al*., 2017; Strępiński *et al*., 2020), and modification of the quantity and organisation of such globules could serve as the developmental mechanism underlying the evolution of the photonic structure in *L. strigocamara*.

Developmentally, the photonic structure also follows a pattern consistent with an evolutionary origin related to cuticle synthesis (Fig. 3). Initially, the cuticle remained thin even when the cell wall (lacking globules) was thick. Over time, globules may migrate through the cell wall and compact against the cuticle. Under this scenario, cuticle size increases until the globular layers begin to form in the cell wall at which point the photonic structure grows layer by layer. During this process, the cell wall lacking globules shrinks as it becomes filled with globular layers. Simultaneously the globular region grows, and the cuticle thickness remains the same. Although this evolutionary story is still speculative at this time, our data are consistent with the possibility that the layered lipids are derived from a modification of cuticle synthesis.

Convergent evolution, in this case, occurs at some scales but not others: phenotypically and morphologically the structure in *L. strigocamara* strongly resembles that in *Viburnum tinus* but chemically these structures are different in ways that may impact their ecological function (see below). Consequently, the scale at which convergence occurs is critical to consider when studying the relationship between structure (in this case, photonic structure) and function (signalling to dispersers). Fruits use a variety of mechanisms to generate a blue colour, including anthocyanins combined with aluminium (e.g. Chenery, 1948), novel pigments (e.g. Iwadare *et al*., 1978) and waxy blooms that create a hue that appears blue to humans, but which reflects strongly in the UV region (Willson & Whelan, 1989). There are also other types of structural colours in fruits/seeds (Vignolini *et al*., 2012a,b,c, 2016) that may have different signalling functions. Whether and how these diverse mechanisms influence plant–animal communication, and whether distinct mechanisms have different signalling functions, is currently unknown.

### Signalling function of structural colour

In *Viburnum tinus* the lipids used to produce the photonic structure are likely to be fatty acids (Middleton *et al*., 2020) that are digestible by birds and therefore may contribute directly to the nutritional content of the fruit, possibly a type of honest signal (Sinnott‐Armstrong *et al*., 2020a). However, honest signalling is highly unlikely in *Lantana strigocamara*. The digestibility of different kinds of lipids varies across birds and mammals, but in general more polymerised lipids tend to be less digestible than short‐chain fatty acids (Place, 1992; Place & Stiles, 1992; Afik & Karasov, 1995; McWilliams *et al*., 2002). Because the lipids in *L. strigocamara*'s photonic structure are polymerised, we do not expect them to contribute to the fruit's nutritional content. Consequently, the lipids likely only contribute to colour signalling and not to the reward signalled by that colour. This is borne out by the lipid content of *L. strigocamara* fruits, which is very low (< 1%) in contrast with the high lipid content of *V. tinus* fruits (*c*. 27%) (Sinnott‐Armstrong *et al*., 2020a).

The colour of the *L. strigocamara* fruits is likely to contribute to successful seed dispersal primarily through being distinctive, rather than serving as an honest signal of nutritional content. There are, however, other potential functions of this type of lipidic structure that may influence its evolution. For instance, the cuticle provides some defence against fungal pathogens (e.g. Yeats & Rose, 2013) and, if this structure is derived from cuticle, it may contribute to defence against fungal pathogens. Alternatively, it may provide enhanced mechanical support to maintain the fruit's shape and/or prevent accidental damage to the fruit skin.

### Implications for seed dispersal

The wide distributions of known structurally coloured plants suggests the possibility that their unusual colour may have contributed to their ability to disperse. Most of the known structurally coloured fruits and seeds are geographically widespread (Sinnott‐Armstrong, 2019). *Viburnum tinus* occurs natively throughout the Mediterranean, and blue‐fruited species of the Tinus clade are native throughout Europe, Asia and some island systems (e.g. Azores, Canaries) (Moura *et al*., 2015). *Elaeocarpus angustifolius* occurs in eastern Australia, and other blue‐fruited species of the genus occur throughout the Indo‐Pacific region as well as in Madagascar and many Pacific islands (Baba, 2013). *Pollia* and *Margaritaria* occur pantropically (Stevens, 2001). *Delarbrea* is the one known exception, with a single blue‐fruited species restricted to the Wet Tropics of Australia (Lee *et al*., 2000).

*Lantana strigocamara* continues this trend. The native range of *L. camara* (one of the species contributing to the horticultural hybrids) extends throughout Central and South America, and hybrid cultivars have become highly invasive in tropical and subtropical regions around the world (Sharma *et al*., 2005; Bhagwat *et al*., 2012; Sundaram *et al*., 2012). *Lantana strigocamara* fruits are widely consumed by native and nonnative frugivores, especially birds, throughout its invasive range (e.g. Chimera & Drake, 2010; Heleno *et al*., 2012; Ramaswami *et al*., 2016). In some key fruit traits (e.g. phenology, morphology), *L. strigocamara* fruit does not differ appreciably from native species (Gosper & Vivian‐Smith, 2006; Ramaswami *et al*., 2016), although our results here point to colour being a very unusual feature of *L. strigocamara* fruits. Furthermore, the long duration of *L. strigocamara* fruiting is unusual (in some parts of the world, *L. strigocamara* fruits are available year‐round) (Corlett, 2005; Gosper & Vivian‐Smith, 2006).

The distinctiveness of the colour of *Lantana strigocamara* fruits may contribute to its invasiveness in tropical regions around the world, although it is probable that other traits – such as the ability to propagate and/or survive in a variety of environments – are also important. The role of distinctive fruit colours in attracting animal dispersers, and whether they are more effective than common colours such as red or black, is unknown but deserves further study. Blue structural colours may provide a strong contrast with the foliage, which is known to be an important factor in removal and seed dispersal (e.g. Cazetta *et al*., 2009). The fruit colour diversity among *Lantana* species makes this a promising system for dissecting the origins and drivers of novel colours.

## Author contributions

MAS‐A, SDS and SV designed the study. MAS‐A, YO and GTvdK collected and analysed data. MAS‐A wrote the initial manuscript draft, and all authors contributed to its revision.

## Supporting information


**Fig. S1** Pre‐ and post‐chloroform extraction transmission electron microscope images of *Lantana strigocamara* epicarp cells.
**Fig. S2** Reflectance of *Lantana strigocamara* fruit under parallel and cross‐polarising filters.
**Fig. S3** Average reflectance spectrum from simulated *Lantana strigocamara* structures.
**Table S1** Protocol for embedding fruit tissue in Epon resin.

**Video S1** Video rotation of tomographic reconstruction of the photonic structure in *Lantana strigocamara*. Bar, 100 nm.
Please note: Wiley Blackwell are not responsible for the content or functionality of any Supporting Information supplied by the authors. Any queries (other than missing material) should be directed to the *New Phytologist* Central Office.

## Data Availability

All data are publicly available at Data Dryad: https://doi.org/10.5061/dryad.98sf7m0m2.
