# Supplementary material for: Convergent evolution of disordered lipidic structural colour in the fruits of Lantana strigocamara (syn. L. camara hybrid cultivar)
Source: New Phytol. 2022 Jun 10;235(3):898–906. doi: 10.1111/nph.18262 (PMC9328138; doi:10.1111/nph.18262)
Supplement: Supplementary file 1 — Fig. S1 Pre‐ and post‐chloroform extraction transmission electron microscope images of Lantana strigocamara epicarp cells. Fig. S2 Reflectance of Lantana strigocamara fruit under parallel and cross‐polarising filters. Fig. S3 Average reflectance spectrum from simulated Lantana strigocamara structures. Table S1 Protocol for embedding fruit tissue in Epon resin. [file NPH-235-898-s001.docx]

***New Phytologist* Supporting Information**

Convergent evolution of disordered lipidic structural color in the fruits of *Lantana strigocamara*

Miranda Sinnott-Armstrong^1,2^, Yu Ogawa^3^, Gea Theodora van de Kerkhof^1^, Silvia Vignolini^1^, Stacey Smith^2^

Article acceptance date: 5 April 2022

**The following Supporting Information is available for this article:**

**Table S1.** Protocol for embedding fruit tissue in Epon resin

**Figure S1.** Pre- and post-chloroform extraction transmission electron microscope (TEM) images of *Lantana strigocamara* epicarp cells

**Figure S2.** Reflectance of *Lantana strigocamara* fruit under parallel and cross-polarizing filters

**Figure S3.** Average reflectance spectrum from simulated *Lantana strigocamara* structures.

Available as a separate file:

**Video S1.** Video rotation of tomographic reconstruction of the photonic structure in *L. strigocamara*

**Table S1**: Protocol for fruit embedding using Epon resin. All steps were performed at room temperature. Samples were placed on a rotator for steps > 10 minutes in length.

| **Step** | **Time** |
| --- | --- |
| Fixative | 1-14 days |
| Buffer wash | 30 min |
| Buffer wash | overnight |
| Osmium | 2 hours |
| Buffer wash | 5 min |
| Buffer wash | 10 min |
| Buffer wash | 10 min |
| Buffer wash | 30 min |
| 30% ethanol | 10 min |
| 50% ethanol | 10 min |
| 70% ethanol | 30 min |
| 90% ethanol | 30 min |
| 100% ethanol | 30 min |
| 100% ethanol | 30 min |
| 100% ethanol | 1 hr |
| 50% ethanol, 50% resin | Evaporate open over night |
| 100% resin | Several hours |
| 100% resin | Several hours |
| 100% resin | Several hours |
| 100% resin | 1 day |
| 100% resin | 1 day |
| Resin + DMP | 12 hours |
| Resin + DMP | 12 hours |
| Polymerization | 24 hours |

**Figure S1.** Reflectance from the fruit under parallel and cross-polarizing filters reveals almost no reflectance in cross-polarization, which indicates that the color is structural rather than (solely) pigmented.

**
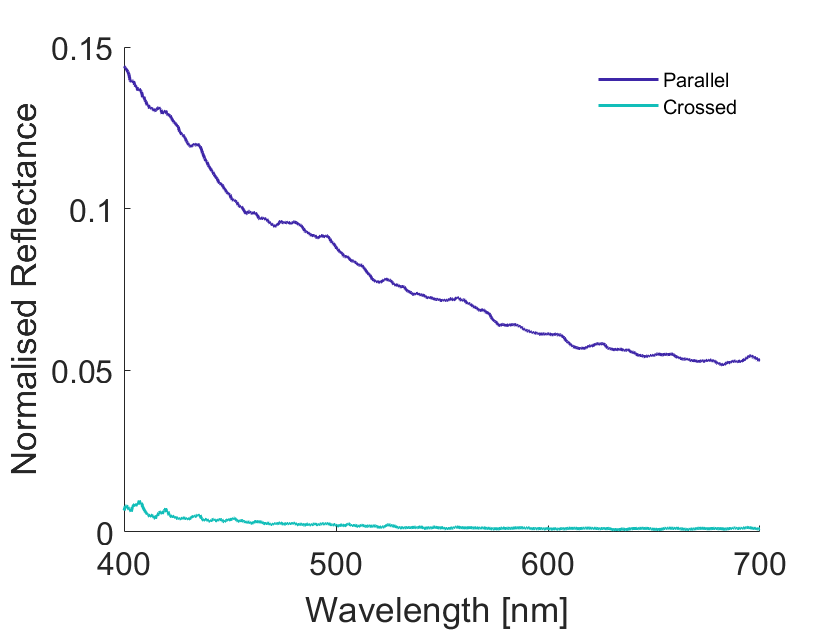
**

**Figure S2.** Reflectance spectrum produced by 2D modeling of the layered photonic structure in *Lantana strigocamara* produces a peak in the UV region of the spectrum, in agreement with optical measurements of reflectance from *L. strigocamara* fruits. The reflectance is slightly UV-shifted relative to that observed in *Viburnum tinus*, which is congruent with measurements of the reflectance of *L. strigocamara* which show a UV-shifted peak relative to measurements of *V. tinus*.

**Figure S3.** Using the technique described in Sinnott-Armstrong (2020b), we exposed *Lantana strigocamara* fruit tissue to chloroform for 1 hour, and imaged before and after. No effect on electron density of the globules was observed, although some reduction in the membrane around the globules did occur. This indicates that the contents of the globules are not chloroform-soluble, but that they may occur within a membrane that is soluble in chloroform. We followed the same procedure for the remainder of the solvents tested.

**
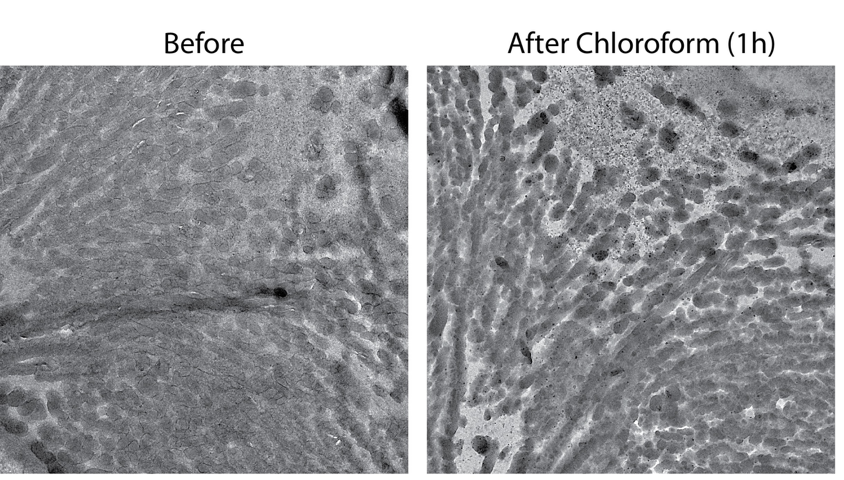
**

**Sinnott-Armstrong M, Vignolini S, Ogawa Y**. **2020b**. Protocol for extraction and electron microscopy visualization of lipids in *Viburnum tinus* fruit using cryo-ultramicrotomy. *STAR Protocols* **1**: 100201.
