# Supplementary figures and images for: Convergent evolution of disordered lipidic structural colour in the fruits of Lantana strigocamara (syn. L. camara hybrid cultivar)
Source: New Phytol. 2022 Jun 10;235(3):898–906. doi: 10.1111/nph.18262 (PMC9328138; doi:10.1111/nph.18262)

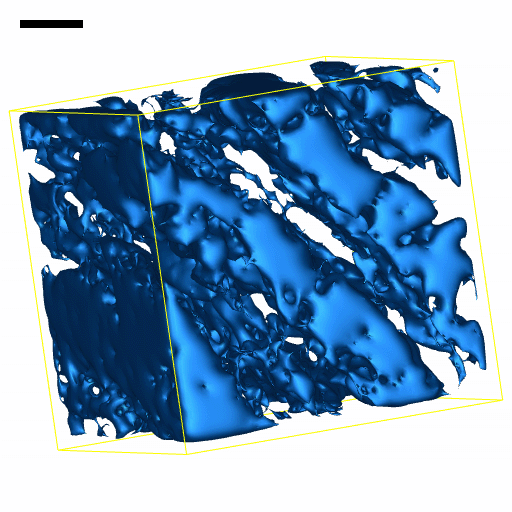

Supplement: Supplementary file 2 — Video S1 Video rotation of tomographic reconstruction of the photonic structure in Lantana strigocamara. Bar, 100 nm. Please note: Wiley Blackwell are not responsible for the content or functionality of any Supporting Information supplied by the authors. Any queries (other than missing material) should be directed to the New Phytologist Central Office. [file NPH-235-898-s002.gif]
